# Synthesis and Biological Evaluation of Thiosemicarbazide Derivatives Endowed with High Activity toward *Mycobacterium Bovis*

**Published:** 2017

**Authors:** Soroush Sardari, Samaneh Feizi, Ali Hossein Rezayan, Parisa Azerang, Seyyed mohammad Shahcheragh, Ghazaleh Ghavami, Azizollah Habibi

**Affiliations:** a *Drug Design and Bioinformatics Unit, Medical Biotechnology Department, Biotechnoloogy Research Center, Pasteur Institute of Iran, Tehran, Iran. *; b *Department of Life Science Engineering, Faculty of New Sciences & Technologies, University of Tehran, Tehran, Iran. *; c *Faculty of Chemistry, Kharazmi University, Tehran, Iran.*

**Keywords:** Thiosemicarbazide derivatives, Biological evaluation, *Mycobacterium bovis*, Anti-tubercular activity, Medicinal chemistry

## Abstract

Thiosemicarbazides are potent intermediates for the synthesis of pharmaceutical and bioactive materials and thus, they are used extensively in the field of medicinal chemistry. The imine bond (-N=CH-) in this compounds are useful in organic synthesis, in particular for the preparation of heterocycles and non-natural β-aminoacids. In this paper the synthesis of some new thiosemicarbazide derivatives by condensation reaction of various aldehydes or ketones with 4-phenylthiosemicarbazide or thiosemicarbazide is reported. This synthesis method has the advantages of high yields and good bioactivity. The structures of these compounds were conﬁrmed by IR, mass, ^1^H NMR, ^13^C NMR, and single-crystal X-ray diffraction studies. All of these compounds were tested for their *in-vitro* anti-mycobacterial activity. The influence of the functional group and position of substituent on anti-bacterial activity of compounds is investigated too. The preliminary results indicated that all of the tested compounds showed good activity against the test organism. The compounds 11 and 30 showed the highest anti-tubercular activity (0.39 μg/mL). This synthesis method has the advantages of high yields and good bioactivity.

## Introduction

Thiosemicarbazone derivatives are of special importance because of their versatile biological and pharmacological activities. Thiosemicarbazides are potent intermediates for the synthesis of pharmaceutical and bioactive materials and thus, they are used extensively in the field of medicinal chemistry. The imine bond (-N=CH-) in this compounds are useful in organic synthesis, in particular for the preparation of heterocycles and non-natural β-aminoacids (1-5). These compounds present a great variety of biological activities ranging from antiviral (6). to anticancer (7). antitumor (8-10). anti-inflammatory and antiamoebic (11-13). activities. The anti-bacterial activity of a series of thiosemicarbazide derivatives has been also reported (14-20).

Tuberculosis (TB) is one of the deadly infectious diseases caused mainly by *Mycobacterium tuberculosis*. This notorious pathogen infects about one-third of the world’s population and is responsible for approximately 2 million deaths worldwide per year (21-23). The World Health Organization (WHO) Fact Sheet on TB estimates that between 2000 and 2020 nearly one billion people will get sick and 35 million will die from TB (24). 

Serious challenges associated with the rising epidemic are multidrug-resistance and the growing number of people co-infected with M. tuberculosis and human immunodeficiency virus (HIV) (25). On the other hand; Bacillus Calmette-Guerin (BCG) is an attenuated strain of *Mycobacterium bovis*, a non-virulent tubercle bacillus very closely related to M. tuberculosis (26, 27). Therefore, *M. bovis* is simpler to use and in less strict biosafety regulations in the lab, hence, it can be used in bioassay instead of M. tuberculosis.

Hence, it is clear that there is an urgent need to develop novel anti-mycobacterial drugs with improved properties such as enhanced activity against multidrug-resistance, reduced toxicity, shortened duration of therapy, rapid mycobactericidial mechanism of action, ability to penetrate host cells and exert anti-mycobacterial effect in the intracellular environment.

In continuation of our research on the development of synthetic methods in heterocyclic chemistry (28-31). and also our drug discovery program (32-34). here we report synthesis of thiosemicarbazide derivatives 3-33 by the condensation of 4-phenylthiosemicarbazide or thiosemicarbazide 1 with various aldehydes (3-30) or ketones (31-33) 2 in MeOH at room temperature and evaluation of their anti-tubercular activity.

## Experimental

Infrared spectra were determined with a Perkin-Elmer 843 spectrometer. Proton nuclear magnetic resonance (^1^H NMR) spectra and carbon nuclear magnetic resonance (^13^C NMR) spectra were determined on a Bruker Avance DRX 500 MHz spectrometer and chemical shifts are reported as δ (ppm) in CDCl_3_ solution (0.05% v/v TMS). 

The chemicals used in this work were purchased from Merck, Fluka and Sigma-Aldrich Chemical Companies. 

*General procedure for the preparation of (3-33)*

To a magnetically stirred solution of substituted thiosemicarbazide (1.0 mmol) in MeOH (30 mL) in a round bottom flask was added a solution of benzaldehyde derivatives (1 mmol) at room temperature. The mixture was stirred for 24 h. After completion of the reaction, the precipitate product was filtered and washed with MeOH (20 mL) and dried at room temperature.

*(E)-1-(3-fluorobenzylidene)thiosemicarbazide (3)*

It was obtained as a white solid; yield: 30%, mp: 185-6 °C (dec.). Anal. Calcd for C_8_H_8_FN_3_S: C, 48.72; H, 4.09; N, 21.30, Found: C, 48.68; H, 4.04; N, 21.37. IR (KBr, cm^-1^): 3394, 3238, 3158, 1602, 1577. ^1^H-NMR (500 MHz, DMSO-d6): δH (ppm) 7.15-7.48 (3H, m, arom), 7.79 (1H, d, ^3^J_HF_=10.5 Hz, arom), 7.99 (1H, s, N=CH), 8.10 and 8.19 (2H, 2bs, NH_2_), 11.45 (1H, s, NH). 

*(E)-1-(3-chlorobenzylidene)thiosemicarbazide (4)*

It was obtained as a white solid; yield: 40%, mp: 195-6 °C (dec.). Anal. Calcd for C_8_H_8_ClN_3_S: C, 44.97; H, 3.77; N, 19.66, Found: C, 45.06; H, 3.75; N, 19.61. IR (KBr, cm^-1^): 3391, 3232, 3152, 1602, 1427. ^1^H-NMR (500 MHz, DMSO-d6): δH (ppm) 7.35-7.97 (4H, m, arom), 7.01 (1H, s, N=CH), 8.14 and 8.20 (2H, 2bs, NH_2_), 11.45 (1H, s, NH). 

*(E)-1-(3-bromobenzylidene)thiosemicarbazide ( 5)*

It was obtained as a white solid; yield: 20%, mp: 204.5-205 °C (dec.). Anal. Calcd for C_8_H_8_BrN_3_S: C, 37.22; H, 3.12; N, 16.28, Found: C, 37.26; H, 3.05; N, 16.31. IR (KBr, cm^-1^): 3386, 3233, 3153, 1602, 1533. ^1^H-NMR (500 MHz, DMSO-d6): δH (ppm) 7.33-7.70 (3H, m, arom), 8.18-8.19 (1H, m, arom), 8.00 (1H, s, N=CH), 8.21 and 8.26 (2H, 2bs, NH_2_), 11.51 (1H, s, NH). ^13^C NMR (75.4 MHz, DMSO): δ 178.1 (C=S), 140.4 (CH=N), 136.6 (Cq Ar), 132.2 (CH Ar), 130.6 (CH Ar), 128.8 (CH Ar), 126.9 (CH Ar), 122.3 (Cq Ar). 

*(E)-1-(4-fluorobenzylidene)thiosemicarbazide* ( 6)

It was obtained as a white solid; yield: 50%, mp: 189.5-191 °C (dec.). Anal. Calcd for C_8_H_8_FN_3_S: C, 48.72; H, 4.09; N, 21.30, Found: C, 48.68; H, 4.07; N, 21.34. IR (KBr, cm^-1^): 3391, 3235, 3157, 1601, 1506. ^1^H-NMR (500 MHz, DMSO-d6): δH (ppm) 7.22-7.25 (2H, m, arom), 7.85-7.88 (2H, m, arom), 8.02 (1H, s, N=CH), 8.04 and 8.19 (2H, 2bs, NH_2_), 11.43 (1H, s, NH).^ 13^C NMR (75.4 MHz, DMSO): δ 177.1 (C=S), 164.6 and 161.3 (CF), 141.0 (CH=N), 135.6 (Cq Ar), 140.6 (CH Ar), 130.8 (Cq Ar), 115.8 (CH Ar). 

*(E)-1-(4-chlorobenzylidene)thiosemicarbazide* ( 7)

It was obtained as a white solid; yield: 13%, mp: 207-208 °C (dec.). Anal. Calcd for C_8_H_8_ClN_3_S: C, 44.97; H, 3.77; N, 19.66, Found: C, 44.95; H, 3.76; N, 19.69. IR (KBr, cm^-1^): 3435, 3281, 3057, 1599, 1466. ^1^H NMR (500 MHz, DMSO-d6): δH (ppm) 7.44-7.84 (4H, m, arom), 8.01 (1H, s, N=CH), 8.07 and 8.23 (2H, 2bs, NH_2_), 11.48 (1H, s, NH). 

*(E)-1-(4-bromobenzylidene)thiosemicarbazide* ( 8)

It was obtained as a white solid; yield: 30%, mp: 194-195 °C (dec.). Anal. Calcd for C_8_H_8_BrN_3_S: C, 37.22; H, 3.12; N, 16.28, Found: C, 37.27; H, 3.10; N, 16.25. IR (KBr, cm^-1^): 3323, 3153, 1593, 1493. ^1^H-NMR (500 MHz, DMSO-d6): δH (ppm) 7.59-7.78 (4H, m, arom), 8.01 (1H, s, N=CH), 8.08 and 8.24 (2H, 2bs, NH_2_), 11.49 (1H, s, NH). ­­­­^13^C NMR (75.4 MHz, DMSO): δ 178.0 (C=S), 140.9 (CH=N), 133.5 (Cq Ar), 131.6 (CH Ar), 129.2 (CH Ar), 123.0 (Cq Ar). 

*(E)-1-(2-nitrobenzylidene)thiosemicarbazide *(9)

It was obtained as a white solid; yield: 82%, mp: 240-241 °C (dec.). Anal. Calcd for C_8_H_8_N_4_O_2_S: C, 42.85; H, 3.60; N, 24.99, Found: C, 42.89; H, 3.58; N, 24.94. IR (KBr, cm^-1^): 1602 (C=N). ^1^H-NMR (500 MHz, DMSO-d6): δH (ppm) 7.61-7.75 (2H, m, arom), 8.02-8.04 (1H, m, arom), 8.43-8.45 (1H, m, arom), 8.14 and 8.40 (2H, 2bs, NH_2_), 8.46 (1H, s, N=CH), 11.74 (1H, s, NH). 

*(E)-1-(3-nitrobenzylidene)thiosemicarbazide ( 10)*

It was obtained as a white solid; yield: 61%, mp: 230-231 °C (dec.). Anal. Calcd for C_8_H_8_N_4_O_2_S: C, 42.85; H, 3.60; N, 24.99 Found: C, 42.75; H, 3.67; N, 25.09. IR (KBr, cm^-1^): 3424, 3157, 2971, 1604, 1515. ^1^H-NMR (500 MHz, DMSO-d6): δH (ppm) 7.67 (1H, t, ^3^J=8.0 Hz, arom), 8.18-8.24 (2H, m, arom), 8.65 (1H, t, ^4^J=1.5 Hz, arom), 8.13 (1H, s, N=CH), 8.31 and 8.33 (2H, 2bs, NH_2_), 11.62 (1H, s, NH).^ 13^C NMR (75.4 MHz, DMSO): δ 178.3 (C=S), 148.3 (Cq Ar), 139.8 (CH=N), 136.1 (Cq Ar), 133.5 (CH Ar), 130.1 (CH Ar), 123.9 (CH Ar), 121.3 (CH Ar). 

(*E)-1-(4-nitrobenzylidene)thiosemicarbazide* *(11*) 

It was obtained as a white solid; yield: 33%, mp: 236-237 °C (dec.). Anal. Calcd for C_8_H_8_N_4_O_2_S: C, 42.85; H, 3.60; N, 24.99, Found: C, 42.81; H, 3.62; N, 24.97. IR (KBr, cm^-1^): 3365, 3141, 1611, 1588. ^1^H-NMR (500 MHz, DMSO-d6): δH (ppm) 8.10-8.22 (4H, m, arom), 8.23 (1H, s, N=CH), 8.28 and 8.42 (2H, 2bs, NH_2_), 11.73 (1H, s, NH). 

*(E)-1-(4-methylbenzylidene)thiosemicarbazide (12)*

It was obtained as a white solid; yield: 40%, mp: 163-164 °C (dec.). Anal. Calcd for C_9_H_11_N_3_S: C, 55.93; H, 5.74; N, 21.74, Found: C, 55.88; H, 5.72; N, 21.76. IR (KBr, cm^-1^): 3240, 3156, 3025, 1598, 1539. ^1^H-NMR (500 MHz, DMSO-d6): δH (ppm) 2.32 (3H, s, CH_3_), 7.22 (2H, d, ^3^J=8.0 Hz, arom), ), 7.68 (2H, d, ^3^J=8.05 Hz, arom), 8.01 (1H, s, N=CH), 7.95 and 8.16 (2H, 2bs, NH_2_), 11.38 (1H, s, NH).^ 13^C NMR (75.4 MHz, DMSO): δ 177.8 (C=S), 142.3 (CH=N), 139.6 (Cq Ar), 131.4 (Cq Ar), 129.2 (CH Ar), 127.2 (CH Ar), 21.0 (CH_3_). 

*(E)-1-(3-methoxybenzylidene)thiosemicarbazide (13)*

It was obtained as a white solid; yield: 37%, mp: 194-195 °C (dec.). Anal. Calcd for C_9_H_11_N_3_OS: C, 51.65; H, 5.30; N, 20.08, Found: C, 51.62; H, 5.31; N, 20.04. IR (KBr, cm^-1^): 3394, 3275, 3109, 1592, 1534. ^1^H-NMR (500 MHz, DMSO-d6): δH (ppm) 3.79 (3H, s, OCH_3_), 6.94-6.96 (3H, m, arom), 7.24-7.43 (1H, m, arom), 8.00 (1H, s, N=CH), 8.06 and 8.22 (2H, 2bs, NH_2_), 11.43 (1H, s, NH).^ 13^C NMR (75.4 MHz, DMSO): δ 177.9 (C=S), 159.5 (Cq Ar), 142.1 (CH=N), 135.6 (Cq Ar), 129.6 (CH Ar), 120.5 (CH Ar), 116.3 (CH Ar), 110.9 (CH Ar), 55.2 (CH_3_). 

*(E)-1-(4-methoxybenzylidene)thiosemicarbazide* *(14)*

It was obtained as a white solid; yield: 30%, mp: 168-169 °C (dec.). Anal. Calcd for C_9_H_11_N_3_OS: C, 51.65; H, 5.30; N, 20.08, Found: C, 51.62; H, 5.27; N, 20.01. IR (KBr, cm^-1^): 3404, 3153, 2970, 1653, 1597. ^1^H-NMR (500 MHz, DMSO-d6): δH (ppm) 3.78 (3H, s, OCH_3_), 6.94-6.96 (2H, m, arom), 7.71-7.74 (2H, m, arom), 7.98 (1H, s, N=CH), 7.91 and 8.11 (2H, 2bs, NH_2_), 11.31 (1H, s, NH).^ 13^C NMR (75.4 MHz, DMSO): δ 177.5 (C=S), 160.6 (Cq Ar), 142.2 (CH=N), 128.9 (CH Ar), 126.7 (Cq Ar), 114.1 (CH Ar), 55.2 (OCH_3_). 

*(E)-1-(2,4-dimethoxybenzylidene)thiosemicarbazide (15)*

It was obtained as a white solid; yield: 60%, mp: 190-191 °C (dec.). Anal. Calcd for C_10_H_13_N_3_O_2_S: C, 50.19; H, 5.48; N, 17.56, Found: C, 50.22; H, 5.49; N, 17.57. IR (KBr, cm^-1^): 3443, 3239, 3183, 1590, 1531. ^1^H-NMR (500 MHz, DMSO-d6): δH (ppm) 3.81(3H, s, OCH_3_), 3.82 (3H, s, OCH_3_), 6.54-660 (2H, m, arom), 8.02 (1H, d, ^3^J=8.5 Hz, arom), 8.31 (1H, s, N=CH), 7.85 and 8.05 (2H, 2bs, NH_2_), 11.30 (1H, s, NH). 

*(E)-1-((furan-2-yl)methylene)thiosemicarbazide* (*16) *

It was obtained as a brown powder; yield: 30%, mp: 135-136 °C (dec.). Anal. Calcd for C_6_H_7_N_3_OS: C, 42.59; H, 4.17; N, 24.83, Found: C, 42.63; H, 4.17; N, 24.82. IR (KBr, cm^-1^): 3411, 3218, 3016, 1609, 1474. ^1^H-NMR (500 MHz, DMSO-d6): δH (ppm) 6.57-6.92 (2H, m, arom), 7.94 (1H, d, ^3^J=20.2 Hz, arom), 7.76 (1H, s, N=CH), 7.56 and 8.15 (2H, 2bs, NH_2_), 11.36 (1H, s, NH). 

*(E)-1-(3-fluorobenzylidene)-4-phenylthiosemicarbazide (17)*

It was obtained as a white solid; yield: 50%, mp: 199-200 °C (dec.). Anal. Calcd for C_14_H_12_FN_3_S: C, 61.52; H, 4.43; N, 15.37, Found: C, 61.54; H, 4.42; N, 15.39. IR (KBr, cm^-1^): 3342, 3138, 2983, 1599, 1510. ^1^H-NMR (500 MHz, DMSO-d6): δH (ppm) 7.17-7.55 (8H, m, arom), 7.95 (1H, d, ^3^J_HF_=10.3 Hz, arom), 8.11 (1H, s, N=CH), 10.16 (1H, s, NH), 11.85 (1H, s, NH). 

*(E)-1-(3-chlorobenzylidene)-4-phenylthiosemicarbazide* *(18)*

It was obtained as a white solid; yield: 30%, mp: 197-198 °C (dec.). Anal. Calcd for C_14_H_12_ClN_3_S: C, 58.03; H, 4.17; N, 14.50, Found: C, 58.00; H, 4.17; N, 14.55. IR (KBr, cm^-1^): 3298, 3139, 3063, 1595, 1509. ^1^H-NMR (500 MHz, DMSO-d6): δH (ppm) 7.17-7.71 (8H, m, arom), 8.09 (1H, s, arom), 8.12 (1H, s, N=CH), 10.17 (1H, s, NH), 11.84 (1H, s, NH).^ 13^C NMR (75.4 MHz, DMSO): δ 176.4 (C=S), 141.2 (CH=N), 139.0 (Cq Ar), 136.3 (Cq Ar), 133.8 (Cq Ar), 130.5 (CH Ar), 129.6 (CH Ar), 128.1 (CH Ar), 127.0 (CH Ar), 126.4 (CH Ar), 126.2 (CH Ar), 125.6 (CH Ar). 

*(E)-1-(3-bromobenzylidene)-4-phenylthiosemicarbazide* *(19)*

It was obtained as a white solid; yield: 75%, mp: 189-190 °C (dec.). Anal. Calcd for C_14_H_12_BrN_3_S: C, 50.31; H, 3.62; N, 12.57, Found: C, 50.27; H, 3.60; N, 12.60. IR (KBr, cm^-1^): 3335, 3296, 3136, 1609, 1551. ^1^H-NMR (500 MHz, DMSO-d6): δH (ppm) 7.22-7.79 (8H, m, arom), 8.29 (1H, s, arom), 8.11 (1H, s, N=CH), 10.23 (1H, s, NH), 11.90 (1H, s, NH).^ 13^C NMR (75.4 MHz, DMSO): δ 176.3 (C=S), 141.1 (CH=N), 139.0 (C_q_ Ar), 136.5 (C_q_ Ar), 132.5 (CH Ar), 130.7 (CH Ar), 129.0 (CH Ar), 128.0 (CH Ar), 127.3 (CH Ar), 126.4 (CH Ar), 125.5 (CH Ar), 122.3 (Cq Ar). 

*(E)-1-(4-fluorobenzylidene)-4-phenylthiosemicarbazide* *(20) *

It was obtained as a white solid; yield: 25%, mp: 179-181 °C (dec.). Anal. Calcd for C_14_H_12_FN_3_S: C, 61.52; H, 4.43; N, 15.37, Found: C, 61.55; H, 4.41; N, 15.38. IR (KBr, cm^-1^): 3315, 3134, 3046, 1601, 1544, 1505. ^1^H-NMR (500 MHz, DMSO-d6): δH (ppm) 7.20-7.55 (7H, m, arom), 7.97-8.00 (2H, m, arom), 8.14 (1H, s, N=CH), 10.15 (1H, s, NH), 11.84 (1H, s, NH). 

*(E)-1-(4-chlorobenzylidene)-4-phenylthiosemicarbazide (21)*

It was obtained as a white solid; yield: 71%, mp: 200-201 °C (dec.). Anal. Calcd for C_14_H_12_ClN_3_S: C, 58.03; H, 4.17; N, 14.50, Found: C, 57.93; H, 4.17; N, 14.55. IR (KBr, cm^-1^): 3309, 3134, 2980, 1595, 1537. ^1^H-NMR (500 MHz, DMSO-d6): δH (ppm) 7.20-7.96 (9H, m, arom), 8.14 (1H, s, N=CH), 10.18 (1H, s, NH), 11.89 (1H, s, NH). 

*(E)-1-(4-bromobenzylidene)-4-phenylthiosemicarbazide* *(22)*

It was obtained as a white solid; yield: 28%, mp: 191-192 °C (dec.). Anal. Calcd for C_14_H_12_BrN_3_S: C, 50.31; H, 3.62; N, 12.57, Found: C, 50.35; H, 3.61; N, 12.55. IR (KBr, cm^-1^): 1602 (C=N). ^1^H-NMR (500 MHz, DMSO-d6): δH (ppm) 7.22-7.79 (8H, m, arom), 8.29 (1H, bs, arom), 8.11 (1H, s, N=CH), 10.24 (1H, s, NH), 11.91 (1H, s, NH).^ 13^C NMR (75.4 MHz, DMSO): δ 176.1 (C=S), 141.5 (CH=N), 139.0 (C_q_ Ar), 133.3 (C_q_ Ar), 131.5 (CH Ar), 129.5 (CH Ar), 128.0 (CH Ar), 126.0 (CH Ar), 125.4 (CH Ar), 123.2 (Cq Ar). MS, m/z (%): 334 (M^+^, 2), 335 (^81^Br, 10), 333 (^79^Br, 10), 199 (^81^Br, 10), 197 (^79^Br, 10), 151 (90), 136 (35), 119 (40), 93 (100), 77 (70), 43 (30).

*(E)-1-(2-nitrobenzylidene)-4-phenylthiosemicarbazide (23)*

It was obtained as a yellow solid; yield: 72%, mp: 199-201 °C (dec.). Anal. Calcd for C_14_H_12_N_4_O_2_S: C, 55.99; H, 4.03; N, 18.65, Found: C, 55.75; H, 4.09; N, 18.51. IR (KBr, cm^-1^): 3341, 3127, 2973, 1692, 1507. ^1^H-NMR (500 MHz, DMSO-d6): δH (ppm) 7.22-7.90 (7H, m, arom), 8.05-8.07 (1H, m, arom), 8.57-8.58 (1H, m, arom), 8.59 (1H, s, N=CH), 10.23 (1H, s, NH), 12.14 (1H, s, NH). ^13^C NMR (75.4 MHz, DMSO): δ 176.4 (C=S), 148.3 (C_q_ Ar), 138.9 (CH=N), 137.8 (C_q_ Ar), 133.3 (CH Ar), 130.5 (CH Ar), 128.6 (CH Ar), 128.3 (CH Ar), 126.0 (Cq Ar), 125.6 (CH Ar), 124.5 (CH Ar). 

*(E)-1-(3-nitrobenzylidene)-4-phenylthiosemicarbazide (24)*

It was obtained as a yellow solid; yield: 57%, mp: 203-204 °C (dec.). Anal. Calcd for C_14_H_12_N_4_O_2_S: C, 55.99; H, 4.03; N, 18.65, Found: C, 55.80; H, 4.00; N, 18.80. IR (KBr) (ν_max_ /cm^-1^): 3228, 3179, 1688, 1525, 1507. ^1^H-NMR (500 MHz, DMSO-d6): δH (ppm) 7.23-8.25 (7H, m, arom), 8.26 (1H, s, N=CH), 8.35-8.37 (1H, m, arom), 8.74-8.75 (1H, m, arom), 10.35 (1H, s, NH), 12.03 (1H, s, NH). 

*(E)-1-(4-nitrobenzylidene)-4-phenylthiosemicarbazide (25)*

It was obtained as a white solid; yield: 33%, mp: 209 °C (dec.). Anal. Calcd for C_14_H_12_N_4_O_2_S: C, 55.99; H, 4.03; N, 18.65, Found: C, 55.16; H, 4.13; N, 18.80. IR (KBr, cm^-1^): 3345, 3315, 2975, 1599, 1582, 1510. ^1^H NMR (500 MHz, DMSO-d6): δH (ppm) 7.23-7.54 (5H, m, arom), 8.20-8.26 (5H, m, N=CH and arom), 10.35 (1H, s, NH), 12.13 (1H, s, NH). MS, m/z (%): 300 (M^+^, 10), 207 (30), 149 (35), 122 (50), 93 (100), 77 (90), 57 (90), 43 (80).

*(E)-1-(4-methylbenzylidene)-4-phenylthiosemicarbazide (26)*

It was obtained as a white solid; yield: 60%, mp: 186 °C (dec.). Anal. Calcd for C_15_H_15_N_3_S: C, 66.88; H, 5.61; N, 15.60, Found: C, 66.18; H, 5.71; N, 15.43. IR (KBr, cm^-1^): 3322, 3298, 2990, 1600, 1551, 1504. ^1^H NMR (500 MHz, DMSO-d6): δH (ppm) 7.19-7.38 (5H, m, arom), 7.56 (2H, d, ^3^J_HH_=7.95 Hz, arom), 7.80 (2H, d, ^3^J_HH_=7.95 Hz, arom), 8.13 (1H, s, N=CH), 10.08 (1H, s, NH), 11.77 (1H, s, NH).^ 13^C NMR (75.4 MHz, DMSO): δ 175.8 (C=S), 143.00 (CH=N), 139.9 (C_q_ Ar), 138.9 (C_q_ Ar), 131.3 (C_q_ Ar), 129.3 (CH Ar), 128.0 (CH Ar), 127.6 (CH Ar), 125.9 (CH Ar), 125.3 (CH Ar), 21.1 (CH_3_). 

*(E)-1-(3-methoxybenzylidene)-4-phenylthiosemicarbazide (27)*

It was obtained as a white solid; yield: 82%, mp: 155-156 °C (dec.). Anal. Calcd for C_15_H_15_N_3_OS: C, 63.13; H, 5.30; N, 14.73, Found: C, 63.43; H, 5.36; N, 14.53. IR (KBr, cm^-1^): 3432, 3286, 3164, 1611, 1599, 1589. ^1^H NMR (5000 MHz, DMSO-d6): δH (ppm) 3.81 (3H, s, OCH_3_), 6.99-7.56 (9H, m, arom), 8.13 (1H, s, N=CH), 10.13 (1H, s, NH), 11.84 (1H, s, NH). 

(*E)-1-(4-methoxybenzylidene)-4-phenylthiosemicarbazide (28)*

It was obtained as a white solid; yield: 57%, mp: 182-183 °C (dec.). Anal. Calcd for C_15_H_15_N_3_OS: C, 63.13; H, 5.30; N, 14.73, Found: C, 62.73; H, 5.44; N, 14.79. IR (KBr, cm^-1^): 3325, 3147, 1607, 1542, 1504. ^1^H NMR (500 MHz, DMSO-d6): δH (ppm) 3.81 (3H, s, OCH_3_), 6.97-7.87 (9H, m, arom), 8.11 (1H, s, N=CH), 10.05 (1H, s, NH), 11.73 (1H, s, NH).

*(E)-1-(2,4-dimethoxybenzylidene)-4-phenylthiosemicarbazide (29)*

It was obtained as a white solid; yield: 70%, mp: 194-195 °C (dec.). Anal. Calcd for C_16_H_17_N_3_O_2_S: C, 60.93; H, 5.43; N, 13.32, Found: C, 61.13; H, 5.43; N, 13.41. IR (KBr, cm^-1^): 3310, 3135, 2942, 1601, 1542. ^1^H NMR (500 MHz, DMSO-d6): δH (ppm) 3.82 (3H, s, OCH_3_), 3.85 (3H, s, OCH_3_), 6.56-8.20 (9H, m, arom), 8.43 (1H, s, N=CH), 9.99 (1H, s, NH), 11.70 (1H, s, NH).^ 13^C NMR (75.4 MHz, DMSO): δ 175.3 (C=S), 162.5 (Cq Ar), 159.3 (Cq Ar), 139.1 (CH=N), 138.7 (Cq Ar), 128.0 (CH Ar), 127.7 (CH Ar), 125.7 (CH Ar), 125.1 (CH Ar), 114.7 (Cq Ar), 106.3 (CH Ar), 97.9 (CH Ar), 55.8 (OCH3), 55.4 (OCH3). 

*(E)-1-((furan-2-yl)methylene)-4-phenylthiosemicarbazide (30)*

It was obtained as a yellow solid; yield: 60%, mp: 183-184 °C (dec.). Anal. Calcd for C_12_H_11_N_3_OS: C, 58.76; H, 4.52; N, 17.13, Found: C, 58.66; H, 4.58; N, 17.03. IR (KBr, cm^-1^): 3291, 3126, 2974, 1618, 1540. ^1^H NMR (500 MHz, DMSO-d6): δH (ppm) 6.66-7.86 (8H, m, arom), 8.08 (1H, s, N=CH), 9.87 (1H, s, NH), 11.84 (1H, s, NH).

*(E)-1-(1-(3-bromophenyl)ethylidene)-4-phenylthiosemicarbazide (31)*

It was obtained as a white solid; yield: 70%, mp: 190-191°C (dec.). Anal. Calcd for C_15_H_14_BrN_3_S: C, 51.73; H, 4.05; N, 12.07, Found: C, 51.57; H, 4.12; N, 11.77. IR (KBr, cm^-1^): 3290, 3140, 2945, 1682, 1540. ^1^H NMR (500 MHz, DMSO-d6): δH (ppm) 2.37 (3H, s, CH_3_), 7.23-8.18 (9H, m, arom), 8.20 (1H, s, N=CH), 10.15 (1H, s, NH), 10.62 (1H, s, NH). 

*(E)-1-(1-(4-bromophenyl)ethylidene)-4-phenylthiosemicarbazide (32)*

It was obtained as a white solid; yield: 55%, mp: 190-191 °C (dec.). Anal. Calcd for C_15_H_14_BrN_3_S: C, 51.73; H, 4.05; N, 12.07, Found: C, 51.43; H, 3.95; N, 12.19. IR (KBr, cm^-1^): 3297, 3261, 1682, 1557. ^1^H NMR (500 MHz, DMSO-d6): δH (ppm) 2.37 (3H, s, CH_3_), 7.22-7.99 (9H, m, arom), 8.25 (1H, s, N=CH), 10.15 (1H, s, NH), 10.62 (1H, s, NH).

*(E)-1-(1-(3-bromophenyl)ethylidene)-4-phenylthiosemicarbazide (33)*

It was obtained as a white solid; yield: 30%, mp: 193-194 °C (dec.). Anal. Calcd for C_9_H_10_BrN_3_S: C, 39.72; H, 3.70; N, 15.44, Found: C, 39.58; H, 3.74; N, 15.53. IR (KBr, cm^-1^): 3410, 3232, 1685. ^1^H NMR (500 MHz, DMSO-d6): δH (ppm) 2.28 (3H, s, CH_3_), 7.56 (2H, d, ^3^J_HH_=8.7 Hz, arom), 7.91 (2H, d, ^3^J_HH_=8.7 Hz, arom), 8.25 (1H, s, N=CH), 8.01 and 8.32 (2H, 2bs, NH_2_), 10.27 (1H, s, NH).

*In-vitro evaluation of anti-mycobacterial activity*

*In-vitro* anti-mycobacterial activity evaluations of the compounds were done by the broth microtiter dilution method) against BCG (1173P2) and ethambutol were used as standard controls. The test compounds were initially dissolved in DMSO to give a concentration of 1 or 2 mg/L. All wells of micro plates received 100 µL of freshly prepared Middle broke 7H9 medium (Himedia, India), except first column. 200 µL of distilled water was added to the first column of 96 well plates to minimize evaporation of the medium in the test wells during incubation. Then 100 µL of test compounds with desired concentrations (1000 or 2000 µL) were added to the wells of the first row (each concentration was assayed in duplicate) and serial dilution was made from the first row to the last. Microbial suspension of BCG (1173P2) (100 µL), which had been prepared with standard concentration of 0.5 Mcfarland and diluted with 1:10 proportion by the distilled water, was added to all test wells. Plates were then sealed and incubated for 4 days at 37 °C. After that 12 µL Tween 80 10% and 20 µL Alamar blue 0.01% (Himedia, India) were added to each test well. The results were assessed after 24 and 48 h. A blue color was interpreted as no bacterial growth, and color change to pink was scored as bacterial growth. Wells with a well-defined pink color were scored as positive for growth. The MIC (minimal inhibition concentration) was defined as the lowest drug concentration, which prevented a color change from blue to pink. Ethambutol (Irandaru, Tehran) were used as positive control and DMSO as negative control.

*Brine shrimp toxicity study*

Brine shrimp lethality bioassay (35-37). (Borowitz *et al*., 1992; Hartl *et al*. 2000; Favilla *et al.,* 2006) was carried out to explore the toxicity of selected compounds with anti-mycobacterial potency. 

Dried cysts (1 g cyst per liter) of brine shrimp (Artemia salina) were hatched in a bottle containing artificial sea water (3.5% (w/v) marine salts/distilled water) at 28–30 °C with strong aeration (flow rate of 7 l/min), under a continuous light regime (1600lux) for 30-35 h. Consequently, the newly hatched brine shrimp larvae (nauplii) were separated from the remaining cysts and collected with a pipette from the lighted side and concentrated in Petri dishes to be immediately utilized for bioassay. Assays were carried out in 24-well flat test plates (Orange Scientifique, Belgium). Acetone100% (Merck, Germany) was utilized for the preparation of different concentrations (1000, 100, 10 and 1 μg/mL) of tested compounds, in triplicates. Each well of treated groups exposed with several concentration of acetone dissolved compounds in the basic salt medium (3.5% (w/v) marine salts /distilled water in addition to poly ethylene glycol (PEG) 6000 (Merck, Germany) 1.2%, while control groups only received basic salt medium. Gallic acid (Merck, Germany) was utilized as positive control; respectively. Following evaporation of vehicle solvent, entire wells introduced with 10 fresh nauplii and put on a shaker with 40 rpm to be aerated at room temperature. After 24 h. the numbers of survivors (larvae were considered dead if they did not exhibit any internal or external movement during several sec of observation) were counted by microscope AC 230V, 50 Hz (Sairan, Iran) and recorded to determine the corrected mortality via following formula: 

Corrected mortality (%) = [(mm_ct_)_t_ - (mm_ct_)_c _/ 100 - mm_ct_)_c_] * 100

Here: 

mm_ct _(mortality of individuals at time t %) = [N_Mm_ (number of died individuals) / N_0_ (initial number of living individuals inevery test well at the beginning of the test)] * 100 

(mm_ct_)_t _= calculated mm_ct _for treated test wells

(mm_ct_)_c _= calculated mm_ct _for control test wells

On the subject of calculated corrected mortality, relevant 50% lethality doses (LD_50_)s with 95% confidence intervals were estimated by Graph Pad Prism 5.0 (2007) for each selected anti-mycobacterial compound (38). 

## Results and Discussion

**Scheme 1:**
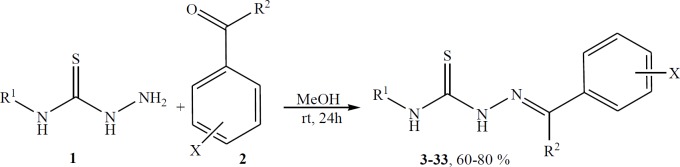
Synthesis of thiosemicarbazide derivatives.

As indicated in Scheme 1, the 1:1 addition reaction of 4-phenylthiosemicarbazide or thiosemicarbazide **1** with aldehydes or ketones occurs smoothly in MeOH at room temperature to produce thiosemicarbazide derivatives 3-33 (39). The structures of the products were deduced from their IR, Elemental Analyzer, mass, ^1^H and ^13^C NMR spectra. The mass spectra of the compound 22 displayed molecular ion peaks at the appropriate m/z values. The ^1^H NMR spectrum of 22 consisted of a singlet for NH group at δ=11.91, another singlet for NH group at δ=10.24, a broad singlet at δ= 8.29 for N=CH group, a singlet for one of the aromatic proton at δ =8.11 and a multiplet for the 8 remaining aromatic ring protons at δ = 7.22-7.79 ppm. The ^1^H decoupled ^13^C NMR spectrum of 22 showed 10 resonances in agreement with proposed structure. Finally, the structure of the product 23 was confirmed unambiguously by single-crystal X-ray analysis (40).

To explore the scope and limitations of this reaction further, we extended our studies to use of various electron-withdrawing and electron-donating substituted aldehydes with 4-phenylthiosemicarbazide and or thiosemicarbazide. As indicated in Table 1, the reactions proceed very efficiently in relatively good yields (3-29). It is interesting to note that when furan-2-carbaldehyde (furfural) was used instead of aryl aldehyde 2 in Scheme 1, the reaction was done and produced 16 and 30 very well. In continue, the reaction of ketones with 4-phenylthiosemicarbazide and or thiosemicarbazide was also checked, and the results are summarized in table 1 (31-33). 

All the synthesized compounds were evaluated for anti-tubercular activity and the results are summarized in Table 1.

As can be seen from Table 1, in compounds 3, the nature of R_1_, R_2_ and X and also the location of X functional group on aromatic ring were investigated on *Mycobacterium bovis* activity. In compounds 3-5 changing of halogen from F to Br in position 3 has same effect (3.9 μg/mL), whilst in position 4 compounds 6-8 the changing of halogen has very different effect on *Mycobacterium bovis* activity (F, Cl, Br = 156: 0.39: 31.25 respectively). It is interesting to note that compound **7** more active than ethambutol which was used as standard controls (0.39 vs 0.75 μg/mL). In compounds 9-11 in which X is NO_2_ (very strong electron-withdrawing group), different location has different effect; in this case meta isomer of these compounds is characterized by higher anti-bacterial activity than their ortho and para isomers (15.6 vs 62.5 and 125 μg/mL).

**Table 1:** Thiosemicarbazide derivatives and their MIC.

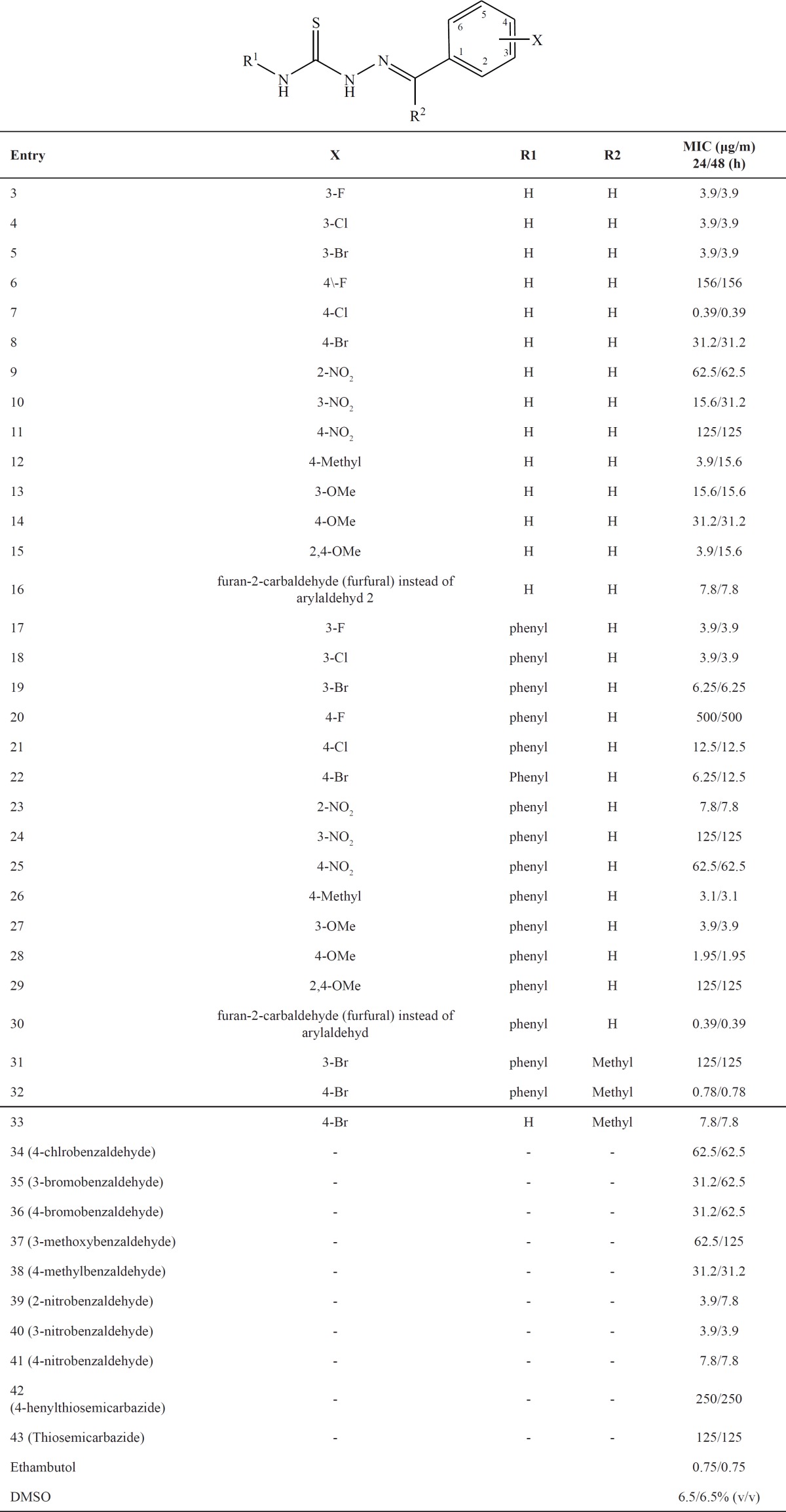

**Table 2:** Data investigated by means of Artemia lethality bioassay

**Derivative**	**LD**_50 _ **(µg/mL)**	**LD**_50_ ** (95% Confidence Intervals)**	**SI**
3	28.47	7.797 to 103.7	7.3
4	4.67	2.033 to 10.76	2.33
13	23.87	8.227 to 69.23	1.52
15	22.15	5.860 to 83.73	2.83
16	52.70	22.86 to 121.5	6.75
17	0.31	0.0001350 to 748.8	0.08
18	1.64	0.03449 to 78.13	0.42
28	12.14	6.054 to 24.33	6.38
30	13.99	2.534 to 77.18	35.87
Gallic acid	23.84	9.780 to 58.13	

No previous literature results no comparison and contrast with previous works [similar molecules showing other activity vs. similar molecules showing similar activity (vs. different other molecules)]. 

About electron-donating substitution (Me or OMe) at positions 3 or 4 and 2, 4 there is not logical trend, but in contrast to electron-withdrawing group (NO_2_), they relatively have more activity (12-15 vs 9-11).

It is important to note that when 4-phenylthiosemicarbazide was used instead of thiosemicarbazide in scheme 1 in the same reaction conditions, the obtained products, 17-30, have similarly activity against *Mycobacterium bovis* in contrast to products 3-16, but in some case more and some case less. 

The important results of table 1 are as follow: **1)** Fluorine in position 4 has low activity (500 μg/mL) and chlorine in position 4 more activity (0.39 μg/mL) than the other halogens with R=H or Ph. In position 3, all of the halogens almost have same activity. **2)** NO_2 _group in positions ortho, meta and para with R=H or Ph no significantly activity differences. 3) In overall with R=H or Ph, electron-donating substitution (Me or OMe) relatively more active than electron-withdrawing group (NO_2_) on aromatic ring. 4) Another important point is that products 16 and 30 which is furan derivatives are active compounds and compound 30 was found to be the most active compound *in-vitro* with an MIC of 0.39 µg/ml against *Mycobacterium bovis* and was 2 times more potent than ethambutol which was used as standard controls (0.39 vs 0.75 μg/mL). 5) Finally products 31-33 in table 1 shows R_2_ = methyl no important effect on compounds activity in contrast to R_2_ = H.

Compounds 34-43 which are starting material, demonstrate different activity against *Mycobacterium bovis*. Among these compounds which have electron-donating or electron-withdrawing substituents on phenyl ring, in general; NO_2_ group more active than the other substituents (39-41 in contrast to 34-38).

Our literature survey showed that a series of thiosemicarbazide derivatives such as thiosemicarbazide and 1,3,4-thiadiazole heterocycles bearing benzo [b] thiophene nucleus, S-alkylisothiosemicarbazone and phenyl/pyridylthiourea compounds was synthesized and evaluated against *Mycobacterium tuberculosis *(41-45). In comparison to reported papers, our results show promising activity (0.39–125 μg/mL vs 6.25, 12.5-100 and 0.14-11.4 μg/mL). 

It may indeed be true that investigating cytotoxicity properties of certain drug candidates is one of the key parameters affecting their fate in lead identification and following that further phases during drug discovery procedures. Recently, a number of toxicity tests have been developed in which the response has been demonstrated in invertebrates. The brine shrimp lethality bioassay (35-38). as one of these assays have the virtue of being economical, reproducible, easy to handle, and environmentally relevant deliberated a practical method for preliminary assessment of toxicity. If given factors as temperature, composition and salinity of the medium and the age of the larvae are considered, a fairly satisfactory repeat-ability is attained. Though, the brine shrimp assay is rather inadequate as regards the elucidation of the mechanism of action (38). It is very practical to assess the toxicity of the natural and synthetic lead compounds. Numerous investigations during recent decades have investigated that the nature of the systems in brine shrimp which respond to drugs appears to be similar to those in mammals that it has ended up with proposing this bioassay for screening biological activities.

In current study, the acute toxicity of anti-mycobacterial compounds, nine of them with uppermost anti-mycobacterial potency as well as considering their diversity, were tested by means of the A. salina short-term bioassay at Pasteur Institute (Tehran, Iran). 

The related LD_50_s demonstrated based on analytical method non-linear regression (dose response inhabitation). Analyzed data showed selected derivatives exposed 50% lethality to nauplii table 1. in doses between 0.31 to 52.70 µg/mL. 

To discover the selectivity of anti-mycobacterial property for each tested compounds, a selectivity index (SI) was calculated by dividing LD_50 _to MIC that the range of calculated SIs was from 0.08 to 35.87. Based on obtained data, it would be obvious that 30, 16 and 28 with SIs 35.87, 6.75 and 6.38 have selective toxicity against *Mycobacterium *in comparison to eukaryotic organism. There would be no doubt that considering SIs for syntactic compounds may lead to introducing a set of novel anti-mycobacterial candidates with minor side effects for further investigations.

## Conclusion

The new thiosemicarbazide derivatives were synthesized by the reaction of various aldehydes and or ketones with 4-phenylthiosemicarbazide or thiosemicarbazide at room temperature. All of these compounds were tested for their *in-vitro* anti-mycobacterial activity. The preliminary results indicated that most of the tested compounds showed good activity against the test organism. The compounds **7** and 30 showed the highest anti-mycobacterial activity (0.39 μg/mL) against *Mycobacterium bovis* (BCG).
